# Prospective workup for the estimation of conceptus dose in fluoroscopically guided procedures

**DOI:** 10.1002/acm2.70037

**Published:** 2025-02-19

**Authors:** Emily L. Marshall, Bryan Schwarz, Megan Glassell, Zheng Feng Lu, Choonsik Lee

**Affiliations:** ^1^ Department of Radiology University of Florida Gainesville Florida USA; ^2^ Department of Radiology University of Chicago Chicago Illinois USA; ^3^ National Institutes of Health National Cancer Institute Bethesda Maryland USA

**Keywords:** conceptus dose, digital phantoms, dosimetry, fluoroscopy, interventional radiology, Monte Carlo modeling, radiation risk, teratogenic risk

## Abstract

**Purpose:**

To present a methodology for the estimation of conceptus dose prior to the completion of a medically required and justified interventional radiology or cardiology procedure.

**Materials and Methods:**

The free of charge National Cancer Institute dosimetry system for Radiography and Fluoroscopy dose calculator tool was adopted to estimate organ absorbed doses through Monte Carlo radiation transport. A procedure was developed for required data collection ahead of the study for dose estimation. This information was used to build the input to the dose calculator tool. Data inclusion and assumption considerations were discussed for final report drafting and communications with the intended interventionalist.

**Results:**

Implementation of this methodology has been used to support pre‐procedural decision making for our institutions interventionalists. Pre‐procedural conceptus dose estimates permitted an educated assessment of the risk‐benefit of the potential radiation exposure to the patient and conceptus against the medical necessity of the procedure. It can also guide the real‐time dose monitoring during the procedure when a maximum permissible cumulative air kerma (CAK) level is determined before the procedure.

**Conclusion:**

A methodology for the estimation of conceptus dose prior to the completion of an interventional fluoroscopy procedure was developed. Due to the prospective nature of this dose estimation methodology, the model relies heavily on professional experience and, when available, quantitative metrics.

## 1 INTRODUCTION

There are many risks associated with providing medical care to pregnant patients, from concerns over contrast agents and anesthesia, to the risk of radiation effects on the developing conceptus.^1^, ^2^ While many of these concerns remain unnavigable by clinical medical physicists, quantification of radiation dose to the conceptus is a task we can take responsibility for. Physicists have the ability to support physicians in their decision‐making process and empower them with quantitative estimates of conceptus dose for a particular procedure prior to the first depression of the fluoroscopy pedal. This information permits an educated assessment and contextualization of the risk‐benefit of the potential radiation exposure to the patient and conceptus against the medical necessity of the procedure.

Potential risks of diagnostic‐level radiation exposures on a conceptus, similar to adults, are binned into tissue and stochastic effects.^3^ Adverse tissue effects are dependent on gestation age, ranging from induction of intellectual disabilities, microcephaly, congenital anomalies, and restrictions on childhood growth.^4^ Threshold doses for each effect vary with the lowest threshold conservatively representing the risk of intellectual disability during the fetal period at doses in excess of 60 mGy.^4^ Numerous challenges exist in defining radiation induced risk of cancer in children following in‐utero radiation exposures. While the effect likely does not abide by a set threshold, it is generally accepted that risks in cancer induction are extremely low when considering doses below 100 mGy.^5^ For this reason, a general consensus to provide continuation in care plans utilizing ionizing radiation in pregnant patients when conceptus radiation dose remains below 100 mGy is maintained by the American College of Radiology (ACR), American Association of Physicists in Medicine (AAPM), International Commission on Radiological Protection (ICRP), and the National Commission on Radiological Protection (NCRP). However, conceptus risk remains at the forefront of clinicians’ minds as they make the decision to move forward with patient care in many cases.

Conceptus dose calculations are commonly made available to physicians, and occasionally patients, prior to or following the completion of a procedure requiring ionizing radiation on a pregnant patient. One of the first published methods of conceptus dose calculation following radiographic exposures was published in 1977 in NCRP Report 54. Continued clinical research efforts on the topic led to iterations in calculation method and refinement in the final dose estimation to the conceptus. However, despite these refinements, clinical calculation of conceptus dose from fluoroscopic procedures is still hampered by two significant limitations: the requirement of post‐procedural data and the uncertainty in the estimate as it is commonly based on fixed technique entrance skin dose values multiplied by percent depth dose curves. Recent advances in phantom morphometry and dosimetry modeling have resulted in open access Monte Carlo based dosimetry platforms. Historically, it has been challenging to estimate the dose of an interventional radiology (IR) procedure ahead of the actual procedure. The nature of such a procedure lends itself to large variation in radiation doses as the physician navigates the case based on feedback they are receiving from the imaging system itself. High‐fidelity Monte Carlo based dosimetry tools in partnership with physician provided anticipated procedural details may be used ahead of IR procedures that could result in a dose of concern to the conceptus, enabling quantitative radiation dose values to drive the risk‐benefit patient‐physician discussions when desired.

The purpose of this work is to develop a methodology for the estimation of conceptus dose prior to the completion of a medically required and justified IR or cardiology procedure.

## 2 METHODS

The prospective nature of this work requires substantial effort from both the intended interventional radiology physician, referred to as the interventionalist throughout the remainder of the paper, and the clinical medical physicist to build a realistic outline of the anticipated patient procedure. This can be accomplished by review of system specific historical trends and interventionalist experience partnered with case impression. The information collected and defined within these first steps will be applied to a software tool to build the radiation conditions and compute an estimate of conceptus dose for a particular female phantom.

### 2.1 Anticipated procedure data collection

The first consideration is a review of patient imaging history with ionizing radiation since the onset of the current pregnancy. Once the gestation age is defined, the type and scope of the procedure and details related to the imaging system employed for the procedure must be considered.

The interventionalist should specify the room in which they anticipate performing the study. Information specific to the fluoroscopy unit assigned should be collected, to include: source to isocenter distance (SID), source to patient skin distance (SSD), dose‐area‐product (DAP) accuracy, x‐ray beam direction, and half‐value layers (HVL) at a typical beam energy setting for the systems added filtration options. Beam energy (kVp) and quality (HVL) are dependent on the protocol, fluoroscopy dose mode, and the imaged patient size. The protocol and fluoroscopy dose mode will be best defined by either the procedure to be performed or feedback from the interventionalist. Information on patient size may be measured directly using prior imaging, or it may be estimated from patient height and weight, or body mass index. In the event no patient specific information is available, average patient data may be taken from institution trends. Once estimates of the protocol, fluoroscopy dose mode, and patient size are identified, physics acceptance testing, annual reports, or a review of institutional radiation dose index monitoring or radiology picture archiving and communication system (PACS) software may be used to extrapolate typical technique selections, notably kVp, of the automatic dose control logic on the unit.^6^ Reports should also have records of the units HVL for the protocol, fluoroscopy dose mode, and kVp once defined.

Once unit specific information is defined or estimated, the x‐ray beam geometry must be considered. Posterior‐anterior (PA) direction is most common in interventional radiology suites. The projection of the x‐ray field‐of‐view (FOV) and the angulation of this projection on the patient during the study will again be best defined by the interventionalist. As part of the data collection process, our site provides the interventionalist with a printed copy of a female adult phantom and requests the x‐ray fields under consideration be outlined on the phantom. In some cases, procedures will require multiple FOVs. When this is the case, the interventionalist is directed to estimate a fraction of total procedure time spent on each. These fields can then be transcribed onto the phantom within the organ dose estimation tool.

**FIGURE 1:**
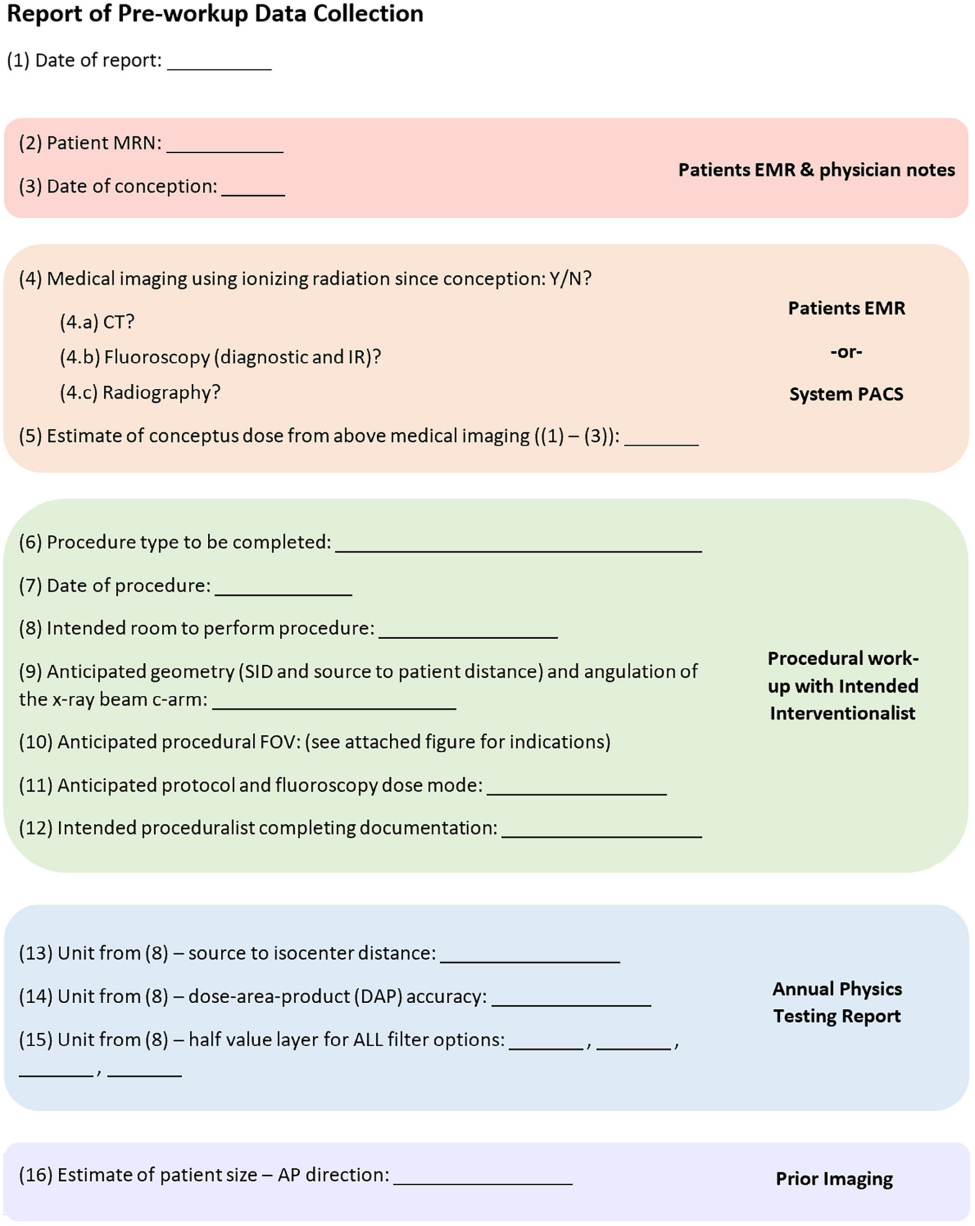
Report form for the pre‐workup data collection needed to support software implementation for estimation of conceptus dose.

Physics annual testing reports for the assigned fluoroscopy unit may be reviewed for dose meter accuracy (i.e. cumulative air kerma (CAK) and DAP meter). Accuracy of the displayed CAK is regulated within ±35% of measured values.^7^ As such, monitoring the accuracy of the specific unit to be used will allow accommodations to be made for deviations between displayed and actual CAK values. A checklist of information to be collected, accompanied by the best source of this information, is provided in Figure 1.

### 2.2 Software implementation

The National Cancer Institute currently offers a dose calculator tool for end users free of charge to estimate organ absorbed doses through Monte Carlo (MC) radiation transport user‐interface.^8^ The tool is named National Cancer Institute dosimetry system for Radiography and Fluoroscopy (NCIRF). It requires input of patient sex and relative age, x‐ray beam data, and x‐ray beam geometry information. These user inputs are then applied within the program to build an MC input file to run simulations for the assessment of organ dose. Since NCIRF does not currently support pregnant female phantoms, the uterine dose reported by the tool serves as the conceptus dose surrogate.

In the context of adult interventional radiology cases, the phantom selection will always be the adult female, likely with the arms down. The software assumes the phantom to be free‐in‐air, as such, the table and pad attenuation are omitted from the calculation. Beam data requires a selection input of energy (kVp) and HVL (mm Al) based on a dropdown list of 25 predefined x‐ray spectra and HVL combinations. To compute organ dose for an energy or HVL combination unavailable within the dropdown options, the calculation must be made for the available spectrum options which encompass the values of interest and interpolation of the doses must then be completed. Inputs for SID, isocenter location (x,y,z), field width and height at isocenter, primary positioner angle, and secondary positioner angle are input as their value in cm. Our site has found success in transcribing the interventionalist defined fields on the phantom to the program based on soft tissue and bony landmarks. An indication of DAP must be inputted into the program. The objective of the dose calculation in this context is to provide the interventionalist insight into the potential conceptus dose should the procedure be performed. As such, there is no post‐procedural reporting of DAP for use in the tool. Our site inputs a value of 100 Gy cm^2^ to the DAP as a simplified value for normalization of organ doses. Finally, the number of simulation particle histories is assigned and the dose calculation can then be performed.

Should the procedure require multiple fields defined by the interventionalist, the dose calculator tool must be run individually for each separate FOV once translated onto the visualized phantom. Each FOV will produce a set of organ doses per DAP input.

### 2.3 Report communications drafting

**FIGURE 2:**
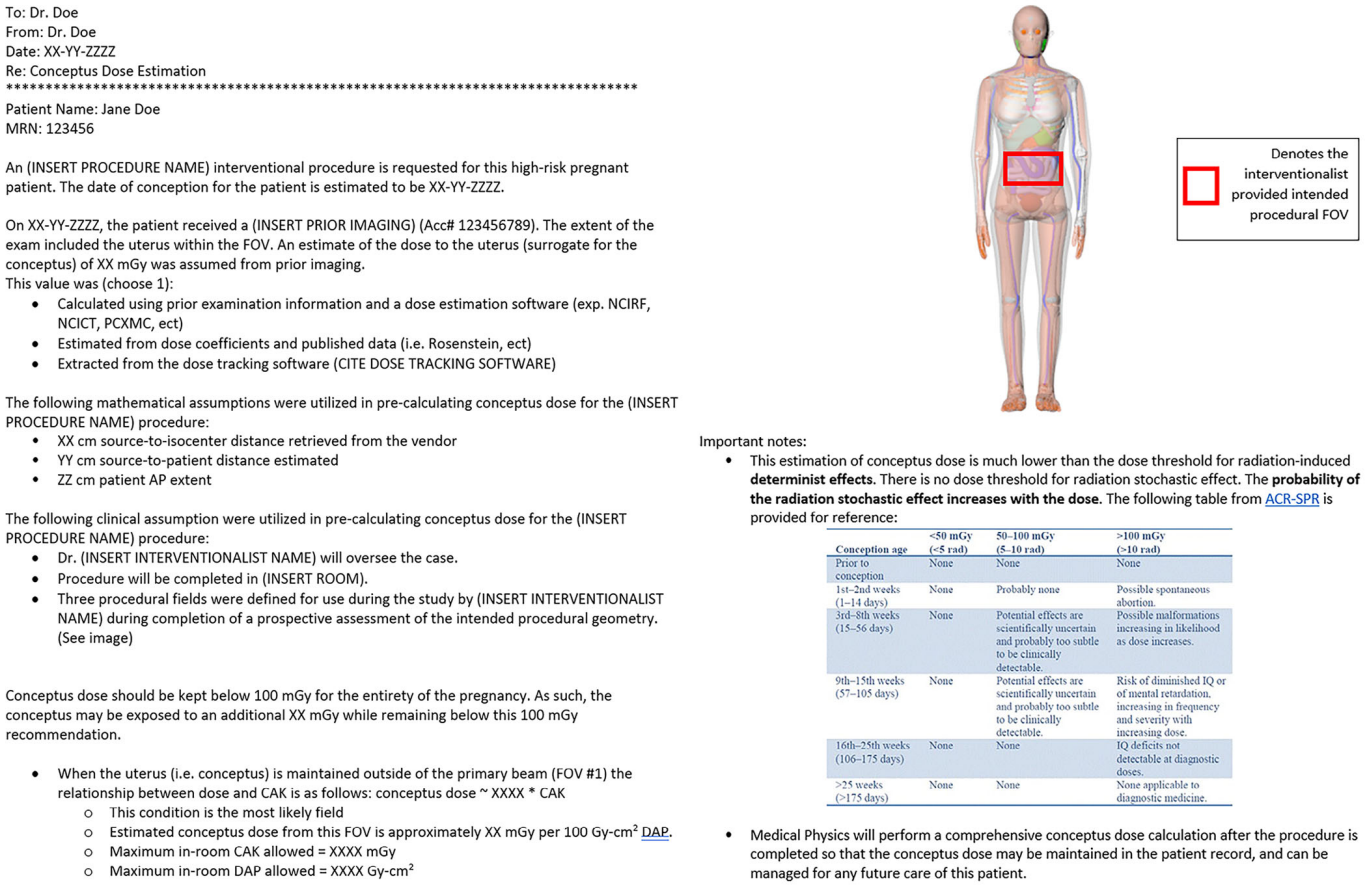
Report structure example for prospective conceptus dose workup.

The prospective conceptus dose report may be presented with three key sections: patient dose history, assumptions associated with the current dose workup, and the dose estimation. A sample report is provided in Figure 2.

The report should begin with a statement of the patient name and procedure under consideration. When available, a statement on the assumed date of conception with an estimate on gestational age should be included. Patient imaging history may be collected from a review of the patient's electronic medical record (EMR) and radiology PACS. The EMR should be comprehensive across departments and as such would include imaging procedures performed beyond radiology that may be missing from the PACS. To estimate dose to the conceptus from prior studies, a retrospective dose calculation should be performed using all radiation dose information available. When dose monitoring software (DMS) systems are available, they may be used to offer estimates of conceptus dose via pregnant female phantoms or uterine dose as a conceptus dose surrogate. In the event that a DMS system is available and the dose information is extracted, the software application and version history should be included in the report. While a DMS offers expeditious access to conceptus dose information, patient and procedural specific dose calculations may also be estimated using previously published dose coefficients or freely‐available dose estimation software tools (NCICT, NICRF).^8^, ^9^, ^10^

As the report is based on a prospective assessment of the procedure possibilities, all assumptions made to arrive at the final dose assessment should be provided as part of the final report. Mathematical assumptions related to geometry may be based on system specification or measurements of patient PA thickness on prior imaging. Clinical assumptions will be those collected during the case review with the interventionalist. Estimates of conceptus dose are highly dependent on FOV geometry, as such, if there is likely to be more than one procedural FOV, each should be separately considered and dose information should be provided for each. Conceptus dose information will be available from the NCIRF software as a function of DAP, but this may be converted to conceptus dose per CAK using the FOV information applied within the software.

Additional items to consider including in the final report for context may be procedural dose reference levels, and up to date information on conceptus risk to ionizing radiation. Utilizing the information provided by the care provider regarding procedure type, a brief literature review may be performed to identify procedural‐specific diagnostic reference levels (DRLs).^11^ Alternatively, if the site participates in the ACR dose index registry (DIR), up to date reference levels may be extracted from the DIR repository.^12^, ^13^ DRLs support efforts toward contextualizing the estimated conceptus dose. Inclusion of up to date radiation related risks may support the provider in their discussions with the patient regarding the planned procedure. This information is available from several sources.^4^, ^14^, ^15^, ^16^

## 3 RESULTS

An example clinical case outlining the implementation of the method itself is presented. A six‐weeks pregnant patient presented to the interventional radiology group at our institution. A splenic artery embolization prior to splenectomy was required at the time of presentation and planned for the following day. The interventionalist approached the physicist and the following work‐up occured.

### 3.1 Anticipated procedure data collection

The patient's EMR was reviewed to determine if any prior imaging had been completed since conception. A CT abdomen/pelvis exam with IV contrast was completed on the patient the day prior with a CTDI_vol_ of 23.8 mGy. The scan was reviewed by the interventionalist and physicists to confirm that the uterus was in‐field during the CT exam. Using the NCICT pregnant female phantom option in the institution's dose tracking software system, an estimate of 34.6 mGy conceptus dose from this CT exam was recorded. Depending upon the technology offered at an institution, the estimate for pre‐procedure imaging conceptus dose may be from a pregnant female phantom calculation, uterine dose calculation surrogate, or an approximation based upon scanner output (e.g., CTDI_vol_). When varying estimate methodology (i.e., pregnant female phantom vs. uterine dose estimate), there was only a small change in the fetal dose estimate (32.6 mGy to 34.6 mGy). The AP extent of the patient was estimated to be 37 cm by measurement from lateral scout images taken during the CT exam.

The procedure was to be completed using a Philips Allura with an annual physics survey completed a few months earlier reporting a HVL of 7.0 mmAl at 80 kVp with 0.4 mm Cu and 1.0 mm Al added filtration for fluoroscopy flavor I mode, and a DAP accuracy of −5%, equating to a correction factor (k) of 1.05. Source‐to‐isocenter distance for this unit was retrieved from the vendor specifications. Discussions with the interventionalist focused on clinical techniques to be utilized during the procedure. There was only one anticipated FOV which would be centered on the spleen along the patient's Z‐axis. The most inferior FOV location possible during the procedure would be approximately mid‐kidney. There was no anticipated use of magnification modes during the procedure.

Physicists instructed the physicians to utilize a low‐dose fluoroscopy mode with the lowest frame rate to minimize conceptus dose. The planned procedure would include minimal fluoroscopy with the conceptus in the primary beam since ultrasound was being utilized for femoral access. The interventionalist informed the physicists that multiple uses of digital acquisitions would be necessary over the spleen. The physicists requested the interventionalist use the lowest frame rate possible when utilizing digital acquisitions and to ensure digital acquisitions would not be acquired if the conceptus was in the primary beam.

### 3.2 Software implementation

The software was run locally on a computer with pre‐approved access. The adult female phantom was selected for use in the NCIRF software version 2.0.20240125. Analysis of the annual physics report demonstrated kVp selections around 80 for the system when operating in low‐dose mode with an attenuation comparable to the patient's PA measurement. The closest match in the NCIRF software was the 80 kVp, 6.38 mmAl bin. Source to patient distance was estimated using the measured 37 cm PA size of the patient, the rooms typical 110 cm source to detector distance (SDD), and an assumed 10 cm working distance between the patient surface and image receptor. This equates to an SSD of 63 cm. The x‐ray field width and height at the patient's surface was back calculated with information on field expectation at the image receptor, SDD, and SSD, using similar triangles. The SSD is used within the program as “source isocenter distance” to simplify the geometry inputs. Placement of the FOV on the phantom in the NCIRF program was completed based on the visual mapped guidance from the interventionalist regarding procedural location. Upper and lower bounds of the FOV were adjusted to ensure they matched the interventionalists direction to map at or above mid‐kidney. Beam geometry was straightforward for this case as the interventionalist outlined a single FOV with a positioner primary and secondary angle of 0 degrees.

The final two items for inclusion in the software were the DAP and particle histories. This calculation was made pre‐procedure to provide an estimate of conceptus dose per DAP. As such, a simple value of 100 Gy‐cm^2^ was inputted for organ dose normalization to DAP. Particle histories of 1,000,000 were run, resulting in uterine dose error less than 10%.

Our case only required one FOV geometry for completion. Should the interventionalist outline multiple FOVs they should indicate an estimate of time spent on each. Each FOV may be used to estimate conceptus dose in NCIRF individually, and weighted by percentage of time spent on each.

### 3.3 Report communications drafting

The above methods were utilized to draft the report with final structure reflected in Figure 2. In our experience, key components of the report include:

- A clear statement of both clinical and physics‐based assumptions.
- A statement regarding the exploratory and estimative nature of the report.
- An estimate of in‐room intra‐procedural CAK/DAP for the full procedure which will maintain the estimated conceptus dose below permissible institutional thresholds.
- When available, literature comparison to studies detailing clinical ranges of CAK/DAP for typical procedures of this type.
- And finally an offer to complete a full conceptus dose calculation post‐procedure using retrospective data.

**FIGURE 3:**
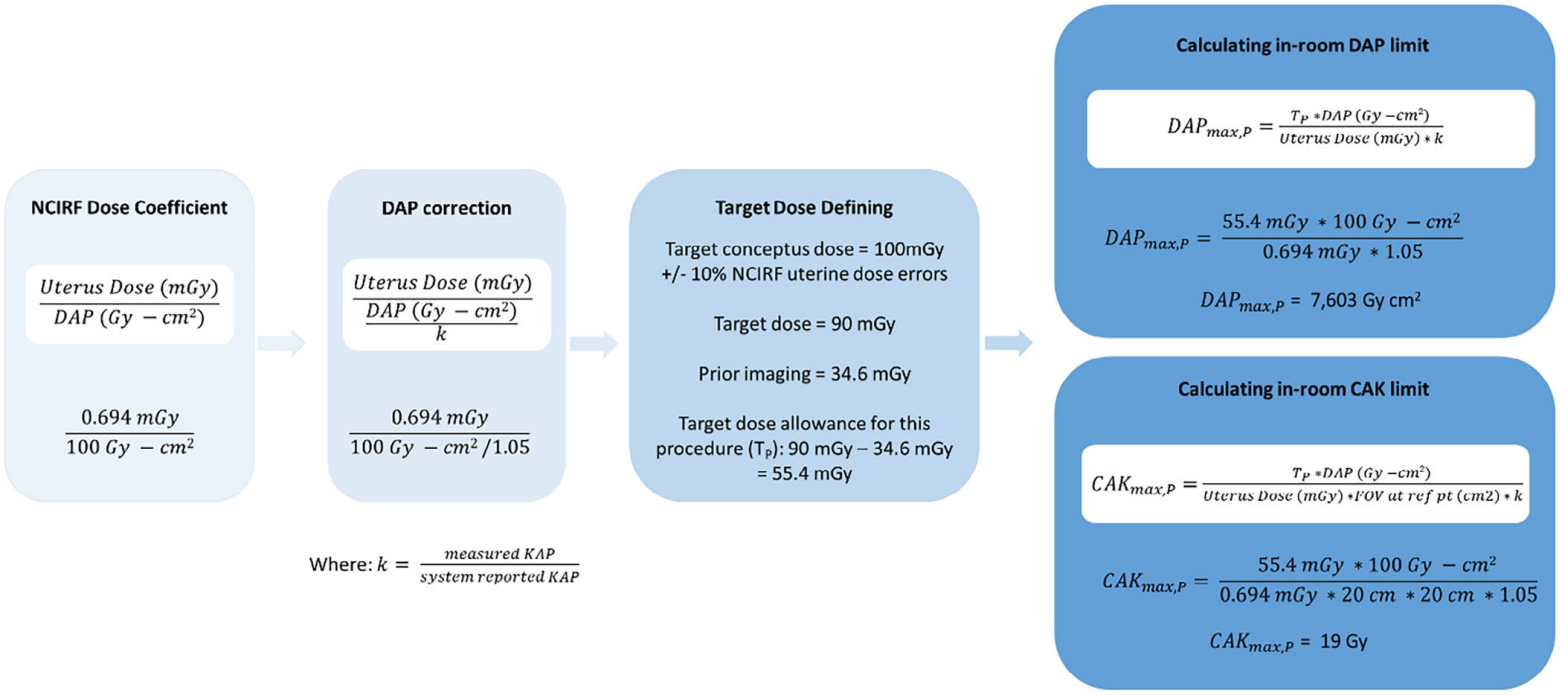
Dose coefficient information produced from NCIRF and the mathematical calculation of DAP and CAK maximum permissible during the procedure. Formulas overlying a white background are general equations, while the remaining content and calculation is specific to the patient case outlined in the results.

Calculation of the patients limiting procedural CAK and DAP is outlined in Figure 3. The conclusion of the report offers these limits to the physician as a numerical guide to monitor CAK and DAP in line with these recommendations during the procedure. For this case, the CAK would reach 19 Gy when the estimated conceptus dose could potentially exceed 100 mGy threshold.

## 4 DISCUSSION

Modeling the anticipated procedure ahead of its occurrence relies on multiple educated assumptions. It is important to build the model based on professional experience and, when available, quantitative metrics. Limitations of the estimate must be well described in the final report and when possible should be discussed between the clinical physicist and the interventionalist in receipt of the report.

Important shifts and form changes occur in pelvic anatomy during pregnancy. By early in the second trimester, the uterus begins expansion and elevation pushing other critical organs superiorly.^17^ The current version of the NCIRF tool does not include a pregnant patient phantom model. For this reason, the proposed methodology is limited in scope as results for patients beyond their first trimester may already demonstrate anatomical variations significant enough from the standard adult female phantom to create unreliable organ dose results. It is relevant to note, many of the adverse tissue effects of high concern are limited to occurrence during the organogenesis and early‐fetal growth period. This limits the period of highest concern in pregnancy to be between two and fifteen weeks. For this reason, we recommend either limiting the dose calculator tool for use in first trimester estimations, or if the information is still requested by the intended interventionalist, providing the dose estimate within the context of potential organ displacement due to late term pregnancy.

## AUTHOR CONTRIBUTIONS

Conceptualization and methodology, Emily Marshall, Zheng Feng Lu, and Bryan Schwarz; Data analysis and presentation, Megan Glassell; Resources and software expertise, Choonsik Lee; Writing, Emily Marshall, and Bryan Schwarz. All authors have read and agreed to the published version of the manuscript.

## CONFLICT OF INTEREST STATEMENT

The authors declare no conflicts of interest.
